# Assessment of the potential of a laser-based device as a *Rhipicephalus microplus* motility inhibitor

**DOI:** 10.1590/S1984-29612025017

**Published:** 2025-03-31

**Authors:** Leonardo Aparecido Lima dos Santos, Velizie Caldarelli Vazquez, Yousmel Alemán Gainza, Ana Carolina de Souza Chagas, Renato Cristiano Torres, Gustavo Felippelli, Alessandro Pelegrine Minho

**Affiliations:** 1 Programa de Pós-Graduação em Ciências Veterinárias, Universidade Estadual Paulista – UNESP, Jaboticabal, SP, Brasil; 2 Instituto de Biociências, Universidade Estadual Paulista – UNESP, Rio Claro, SP, Brasil; 3 Empresa Brasileira de Pesquisa Agropecuária – Embrapa Pecuária Sudeste, São Carlos, SP, Brasil; 4 Empresa Brasileira de Pesquisa Agropecuária – Embrapa, Gerência-Geral de Tecnologia da Informação, Parque Estação Biológica – PqEB, Brasília, DF, Brasil; 5 Faculdade de Ciências Agrárias e Veterinárias, Universidade Estadual Paulista – UNESP, Jaboticabal, SP, Brasil

**Keywords:** Tick, physical control, ectoparasite, resistance, Carrapato, controle físico, ectoparasita, resistência

## Abstract

*Rhipicephalus microplus* is an ectoparasite responsible for causing economic losses in livestock farming totalling approximately $3.24 billion dollars per year. The main control method involves the use of chemical acaricides. However, the incorrect and intensive use of these chemicals has led to an increasing number of reports of resistance to acaricides. Therefore, the objective of this study was to develop and evaluate a laser prototype with a voltage of 5 V and power of 1000 mW to reduce the development of *R. microplus*. The methodology evaluated did not result in a high mortality rate; therefore, it was necessary to carry out a larval migration test. To carry out the test, 3 treatments were evaluated in triplicate (negative controls H_2_0, 60% ethanol and positive control), with 20 larvae were evaluated for each replicate of the treatments, which resulted in an increase in the percentage of migration from 2.5% and 3.2% to above 94.9% and 93.5% in the negative controls, while in the positive control group there was no showed a significant change in migration, reaching close to 100%. This study demonstrated that physical control caused damage to ectoparasite locomotor structures and could affect the parasite's life cycle.

## Resumo

*Rhipicephalus microplus* é um ectoparasita responsável por causar perdas econômicas na pecuária, totalizando aproximadamente US$ 3,24 bilhões de dólares por ano. O principal método de controle envolve o uso de acaricidas químicos. Contudo, o uso incorreto e intensivo desses produtos químicos tem levado a um número crescente de relatos de resistência a acaricidas. Portanto, o objetivo deste estudo foi desenvolver e avaliar um protótipo laser com tensão de 5 V e potência de 1000 mW para reduzir o desenvolvimento do *R. microplus*. A metodologia avaliada não resultou em elevada taxa de mortalidade, portanto, foi necessária a realização do teste de migração larval. Para a realização do teste foram avaliados 3 tratamentos em triplicata (controles negativos H_2_0, etanol 60% e controle positivo), sendo avaliadas 20 larvas para cada réplica dos tratamentos, que resultou no aumento da porcentagem de migração de 2,5% e 3,2% para acima de 94,9% e 93,5% nos controles negativos, enquanto o grupo controle positivo não apresentou alteração significativa na migração, ficando próximo de 100%. Este estudo demonstrou que o controle físico causou danos às estruturas locomotoras dos ectoparasitas, podendo afetar o ciclo de vida parasitário.

## Introduction

The cattle tick is considered one of the most worrying ectoparasites of cattle in Africa, Australia and Latin America and is highly relevant as a vector of *Babesia bigemina*, *Babesia bovis*, *Anaplasma marginale* (agents of Bovine Parasitic Disease - BPD) and *Borrelia theileri* in South America, south and central regions (Taylor et al., 2007; Pereira et al., 2008; Catto et al., 2010; Estrada-Peña, 2015; Marques et al., 2020).

In addition to the transmission of pathogens such as BPD, adult and immature forms of hematophagy cause anaemia, weight loss, stress, decreased immunity, decreased productivity and, occasionally, death in animals (Faza et al., 2013; Lopes et al., 2013). Furthermore, these lesions predispose individuals to the development of myiasis, which negatively affects the quality of the leather (Guglielmone et al., 1999).

Its control involves spending not only on medicines but also on time, facilities and labour for treatment, which indirectly generates economic losses for livestock farmers. According to Grisi et al. (2014), in Brazil alone, losses resulting from this parasite total approximately $3.24 billion dollars per year. Other researchers estimated these impacts for other regions of globe, AUS$175 million per annum in Australia, US$787.63 million/annum in India, US$573.61 million/annum in Mexico, US$308.144 in Uganda, (Playford et al., 2005; Ocaido et al., 2009; Rodriguez-Vivas et al., 2017; Singh et al., 2022).

The main method used to control *R. microplus* is still the application of commercial synthetic acaricides, with the period of residual protection and the efficiency of the product on the target population being essential factors for success (Pereira, 2009; Corrêa et al., 2015).

A synthesis of selected records on the global status of acaricide resistance, in particular for *R. microplus*, is provided by Abbas et al. (2014) and Rodriguez-Vivas et al. (2018). In Brazil, there has been an increase in the number of scientific reports about the resistance of the tick *R. microplus* in recent decades (Raynal et al., 2013; Cruz et al., 2015; Higa et al., 2015), resulting in the spread of resistant tick populations throughout the nation (Higa et al., 2015). In several states, mainly in the southern and southeastern regions of the country, populations resistant to up to eight classes of acaricides and their associations are widely diagnosed (Higa et al., 2015), including the first case of resistance to fluazuron (Reck et al., 2014).

The choice and correct use of these products, as well as changing the product, when necessary, are preponderant factors in obtaining the expected results, as the development of resistant tick populations has historically occurred after a period of use of most tick acaricide released in the country market (Gomes, 2009). According to Furlong (2005), the mechanisms generally used by resistant ticks to survive the application of acaricide include a reduction in the rate of penetration of the product, changes in metabolism, storage and elimination of the chemical; and changes in the site of product action.

Even so, chemical tick killers still represent the best option for livestock farmers, as they are more easily found commercially and present satisfactory results in combating ticks. These materials are generally commercially available in spray, pour-on or injectable forms (Furtado et al., 2013). Regardless of the alternative selected to control parasites, it is advisable to combine several methodologies to achieve success (Pereira et al., 2010), that is, to implement integrated parasite control.

Given the importance of the *R. microplus* tick in Brazilian livestock farming and, given the constant difficulty in controlling this ectoparasite due to resistance to available active ingredients, new studies are necessary, especially on alternatives that help in control, reducing the burden of the parasite on animals and the environment and, consequently, reducing the economic losses caused by them. In this context, it is necessary to develop new methods to control this tick.

The use of laser radiation represents a promising methodology for controlling agricultural and medical pests (Keller et al., 2016). Previous work has demonstrated that exposure to low-power blue light for several hours can incapacitate mosquitoes and fruit flies (Hori et al., 2014). To penetrate parts of the tick's exoskeleton using a laser, it is necessary to increase the temperature at the point of contact and produce a thermal reaction to destroy vital organs.

The integument is an organ that is directly involved in the biological success of ticks due to its protection against mechanical injuries, regulation of water balance, physiological aspects and interaction with the other organs (Anderson & Magnarelli, 2008; Kaufman, 2014). The integument is formed by the epidermis, which promotes the secretion of the cuticle. The tick has two cuticular layers, the outermost, thin and flexible epicuticle and the innermost and thickest procuticle (Kaufman, 2014). The procuticle is divided into two sublayers, the exocuticle and the endocuticle, which are basically composed of chitin and protein (Aguiar, 2020).

Therefore, the objective of this study was to evaluate *in vitro* a new methodology based on physical control using laser-emitting equipment with a power of 1000 mW for the control of immature forms of *R. microplus*.

## Materials and Methods

### Maintenance of *Rhipicephalus microplus* colonies

Adult females were randomly collected from eight dairy host cattle kept without acaricide treatment for at least 30 days, belonging to the colony maintained at the Brazilian Agricultural Research Corporation - Embrapa Southeast Livestock (CPPSE). The females free of drug residues were sanitized, placed in Petri dishes and in an incubator at the Veterinary Parasitology Laboratory (±27 °C; RH > 80%) to perform oviposition. Subsequently, after 18 days, the egg mass was weighed and transferred to adapted and sealed syringes for larval hatching for a period of 15 days. Following, *R. microplus* larvae of 7 to 21 days of age were used to carry out the different tests.

### Laser prototype

**Figure 1:**
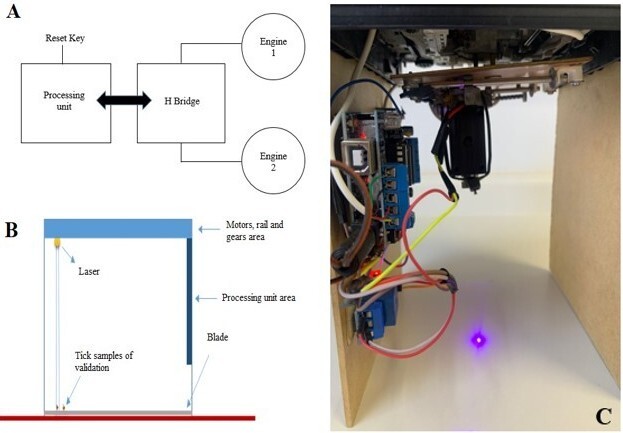
A) Equipment operation diagram; B) Conceptual image of the prototype; C) Real image of the prototype.

Source: Author.

The experimental equipment (Figure 1A) was designed to consist of a processing unit a motor control unit (H-bridge), stepper motors (Engine 1 & 2) and a laser (Figure 1B).

The processing unit is constructed of a prototype development board called Arduino, whose main function is to control the motors and laser firing using embedded software (Figure 1C).

The H Bridge is a type of electronic circuit used to control a direct current (DC) electric motor. Through the control ports, the motor power can be turned on and off. In addition, the direction of rotation of the engine can be changed using keys.

To penetrate parts of the tick's exoskeleton using the laser, it was necessary to adjust the power to approximately 1 W at the point of contact for 100 ms (scanline laser) and produce a thermal reaction to destroy the tick's vital organs and avoid harming the host animal's well-being.

The equipment used was a prototype developed by CPPSE, using maximum power that, theoretically, does not cause damage to the cattle's integumentary system (no animal tests were carried out). A blue‒violet laser with a wavelength of 405 nm, voltage 5 V and power 1000 mW was used in an automated chamber that delimits an area of 4.5 cm and a target height of 4.5 cm.

**Figure 2:**
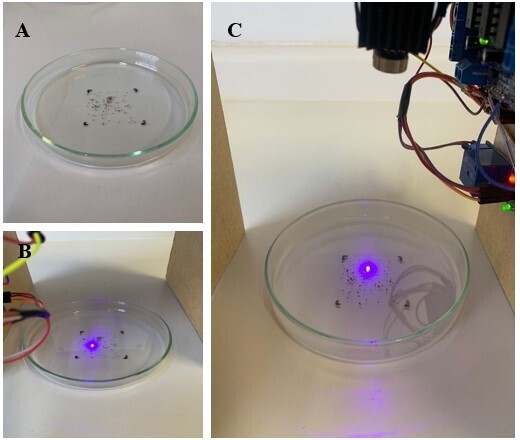
A) Larvae were added to a Petri dish; B) Larvae exposed to laser equipment; C) scanning of larvae exposed to the equipment.

Source: Author.

To evaluate the prototype, 20 *R. microplus* larvae were added to a Petri dish (Figure 2A) in the delimited area. The prototype was contacted only once on a white background (Figure 2B) during the 45-second period (time duration of the scan in the delimited area). We then made contact a second time on a white background, within a time limit of approximately 5 seconds after the first contact (Figure 2C).

Subsequently, the test was performed on a dark background to observe whether the color of the background (dark or light) would affect the dispersion of the laser light, altering the results obtained. Double-sided adhesive tape was glued around the plate to prevent the larvae from escaping. The plates were then exposed to the laser equipment to begin scanning.

**Figure 3:**
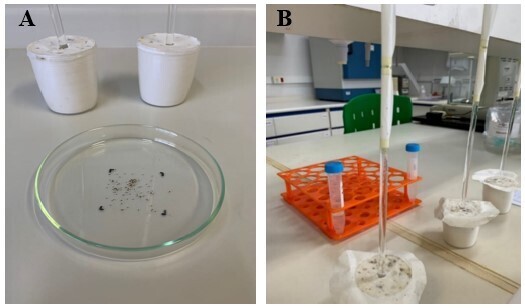
A) Larvae after contact with the laser; B) Test for evaluating inhibition of larval migration.

Source: Author.

Immediately after being exposed to the laser (Figure 3A), the larvae were subjected to the larval migration test (Chagas & Rabelo, 2012) to assess whether there was damage to the sensory and/or locomotor structures (legs and Haller's organ) by evaluating the motility of the larvae in the glass rod (Figure 3B).

### Larval migration inhibition assessment test

**Figure 4:**
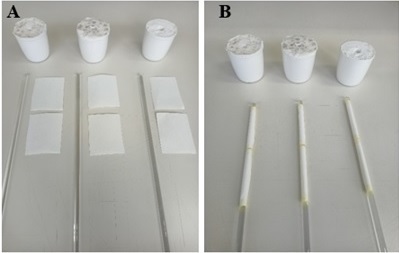
A) 5 cm × 5 cm filter papers, glass rod and plaster support used to perform the larval migration test; B) Filter papers inserted into the rod later called Zone 1 and Zone 2.

Source: Author.

The test was performed according to Chagas & Rabelo (2012), with minor adjustments. Two 5 cm × 5 cm filter papers were cut (Figure 4A) and fixed to the top of a glass rod, being called Zone 1 and Zone 2 (Figure 4B), after which the rod was inserted into a plaster support to fix it, in which we used three rods per treatment (group).

Two groups were created for negative controls (H_2_O and 60% ethanol), and one was created for the positive control (an aqueous extract derived from the *Croton sonderianus* plant, diluted in 60% ethanol at a concentration of 20 µg/mL); the positive control group using *C. sonderianus* extract was evaluated in previous research (unpublished data), in which the migration of infected *R. microplus* larvae was inhibited above 95%, i.e., percentage of migration inhibition ≥ 95%.

The initial hypothesis was to evaluate whether larvae exposed to laser equipment presented possible damage to sensory and/or locomotor organs according to the following calculation:

Migration inhibition percentage = [(number of larvae on nonimpregnated paper Area 2 * 20)/(number of larvae on nonimpregnated paper Area 2 + number of larvae on impregnated paper Area 1)].

For each methodology, analyses were performed in triplicate, in which we added 1 mL of the respective solution in Zone 1, negative controls (H_2_O and 60% ethanol) and positive control (*C. sonderianus*), while we added the larvae (20 per treatment) subjected to the equipment in Zone 2. (Note: treatments were kept at a minimum distance of 2 m.)

After a period of 15 minutes of exposure to the test solution, the larvae were quantified with the aid of a vacuum pump with an adapted tip (1000 µL).

### Statistical analysis

The test results were subjected to one-way analysis of variance (ANOVA) followed by the Tukey–Kramer test to determine the % migration of *R. microplus* larvae. All analyses were performed with R v.4.2.2 software (R Core Team, 2020).

## Results and Discussion

Compared to vertebrates, invertebrates have been studied little in the field of radiobiology or laser therapy. Research has focused mainly on methods for sterilization and eradication of some pest species through the sterile insect technique (Alphey, 2002; Dyck et al., 2021; Argilés-Herrero et al., 2023). This work, for the first time in Brazil, aimed to expand the impacts of laser exposure on less studied species, such as ticks, as well as elucidate the effects on more subtle parameters that may be relevant for the control of these ectoparasites in the environment such as the Atlantic Forest and Cerrado Biomes.

After quantifying the *R. microplus* larvae exposed to the laser and subjected to the migration test, we obtained the following results (Table 1).

**Table 1:** Means ± standard error of the percentage of inhibition of larval migration was evaluated by a migration test after the larvae were subjected to different methods (without laser, 1x laser or 2x laser) and different treatments (negative controls of H_2_O and 60% ethanol and a positive control of *Croton sonderianus*).

**Submitted methods**	**Treatments**	**% migration inhibition**
Without laser	Water	2.5± 4.3^Aa^
Without laser	Ethanol 60%	3.2± 3.3^Aa^
Without laser	*Croton sonderianus*	98.7± 2.3^Aa^
1x Laser-on-White Background	Water	95.4± 4.8^Ba^
1x Laser-on-White Background	Ethanol 60%	96.2± 3.9^Ba^
1x Laser-on-White Background	*Croton sonderianus*	100 ± 0.0^Ba^
2x Laser-on-White Background	Water	96.3± 6.4^Ba^
2x Laser-on-White Background	Ethanol 60%	95.4± 5.7^Ba^
2x Laser-on-White Background	*Croton sonderianus*	98.3± 2.9^Ba^
1x Laser-on-Dark Background	Water	94.9± 4.5^Bb^
1x Laser-on-Dark Background	Ethanol 60%	93.5± 5.4^Bb^
1x Laser-on-Dark Background	*Croton sonderianus*	98.5± 2.6^Bb^
2x Laser-on-Dark Background	Water	97.1± 2.6^Bb^
2x Laser-on-Dark Background	Ethanol 60%	94.3± 4.7^Bb^
2x Laser-on-Dark Background	*Croton sonderianus*	98.2± 3.0^Bb^

The capital letters compare the different methods (without/with laser), and the lowercase letters compare the different backgrounds (white/dark).

The larvae exposed to the laser did not die but lost the ability to move when subjected to the migration test (P≤0.001), and thus were unable to migrate to the region of Zone 1 according to their behavior in the negative geotropism of the infectious stage (Fernandes et al., 2011). Thus, exposure to a low-intensity laser significantly inhibited the motility of *R. microplus* larvae. Additionally, the use of laser-based technology for pest control is much more common than in veterinary parasite control. Gaetani et al. (2021) analysed the lethal dose necessary to kill 90% of two agricultural pests (aphids - *Acyrthosiphon pisum* and *Rhopalosiphum padi*); they showed that irradiating insects at an early stage is crucial to reducing the lethal dose, so laser irradiation is almost always lethal, but can also cause insect stunting and/or reduce female fecundity.

The use of laser radiation represents a promising methodology for the control of agricultural and medical pests through the emission of light from radiation stimulation (Keller et al., 2016). The use of physical control for insects is common and was initiated by Hunter (1912), who used X-rays to treat *Sitophilus oryzae*, but did not obtain satisfactory results due to the low energy used in his work. Muller (1927) evaluated ionizing radiation in *Drosophila melanogaster* flies, but only on the sterility of the evaluated specimens. As in the study mentioned above (Hunter, 1912), the evaluation of our laser prototype in *R. microplus* larvae did not reveal significant mortality due to the low radiation of the laser used; however, it was possible to observe that the larvae subjected to the prototype presented difficulty in locomotion when subjected to the larval migration test. Wilde (1965), when evaluating laser pulses in larvae of *Dermestidae* spp. and *Trogoderma versicolour*, observed that the larvae lost the ability to move, similar to the results presented. According to Gaetani et al. (2021), a global strategy based on the destruction of pests by laser can be considered solid.

By analysing the results of the present study, it was possible to observe that the larvae not exposed to the laser in the negative control groups (H_2_O and 60% ethanol) migrated to the Zone 1 region in accordance with their behaviour in the infective stage, showing migration inhibition of 2.5% and 3.2%.

After contact with the prototype once, the larvae exhibited an immobile appearance on both backgrounds (white and dark). Larvae in the negative control group inhibited 95.4% and 96.2% on a white background, 94.9% and 93.5% on a dark background, during the migration test, the larvae remained in Zone 2, where they were deposited. In the same way, previous study by Hori et al. (2014) demonstrated that exposure to low-power blue light for several hours can disable the motility of *D. melanogaster*. In the present trial, compared to those in the control groups (negative and positive controls), after contact with the laser, the larvae in the control group exhibited damage to their locomotor structures (legs), lost the ability to migrate, and lacked the ability to migrate from Zone 2 to Zone 1.

Due to the larvae showing reduced locomotion but still being alive, a laser scan was performed twice to assess the mortality rate of the larvae. The results were similar when the laser was applied once or twice, with no significant difference. There was no mortality in any application, all the larvae remained alive, and again, in both groups (negative and positive controls), the larvae presented difficulty in locomotion, with migration inhibition percentages of 96.3% and 95.4%, respectively, on a white background and 97.1% and 94.3%, respectively, on a dark background. In studies carried out by Bakri et al. (2005), who evaluated doses of radiation as a tick control strategy for which temperatures were not high enough to affect the survival of the arthropods evaluated, light from Light-emitting diodes (LEDs) was not shown to potentially cause mortality.

Although it was not the main theme of this work and was used only to confirm the efficacy and viability of the test, the compound used in the positive control group, an aqueous extract of *C. sonderianus*, also inhibited the migration of the larvae evaluated, which remained in Zone 2, presenting an inhibition of 98.7% without contact with the laser (the larvae had motility, but did not move to Zone 1), subsequently presenting migration inhibition of 100% and 98.5% in contact with laser 1x, 98.3% and 98.2% in contact with laser 2x, as expected there was no change when evaluating with and without contact with the laser. Studies reported in the literature for essential oils from *C. sonderianus* leaves show that these oils are composed of different sesquiterpenes (Castro et al., 2023). According to Enan (2001), regardless of the specific mode of action of essential oils and their constituents, they play an important role in the effectiveness by maximising the penetration of bioactive agents through the arthropod cuticle, increasing the effectiveness of the acaricidal potential arising from the sesquiterpenes existing in the *C. sonderianus* oil.

The results showed that the laser has the potential to disable locomotor structures (legs). However, the relationship between arthropods and low-power laser tolerance requires further investigation to determine how to utilize blue light irradiation for pest control. According to Araujo & Spencer (2017), blue light irradiation can be useful for controlling several insect pests. However, because the effective wavelengths of blue light are species specific, multiple wavelengths (or broad-spectrum blue light) are needed for simultaneous control of multiple species.

The sensory capacity of these larvae cannot be assessed, as a microscopic study of the physiological damage caused by the laser beam to the structures of the larvae (Haller's organ) is necessary since larval motility was inhibited. According to Klompen & Oliver (1993), Haller's organ includes a large set of different types of chemoreceptive sensors in different species of ticks and is an important target organ for future evaluations of the effect of using low-power lasers to control ticks in production animals. This indicates the need for additional morphological and physiological studies expanding the area of knowledge on physical control in ticks of veterinary interest.

An additional consideration for tick control is the cost of the method in relation to the costs of the diseases that these ectoparasites transmit. The worldwide economic loss due to tick-borne diseases and tick infestations, along with the costs of vaccination and acaricide treatment, is estimated at billions of dollars annually (Jongejan & Uilenberg, 2004). In line with studies on the physical control of diseased vector insects and mites, there is strong public resistance to the widespread dissemination of acaricides in the natural environment, and in many areas, local and/or regional regulations prevent such use. In contrast, once laser treatment is implemented, brief visits to establishments are needed, as the treatment is limited by the tick's seasonal activity period, but mainly because of the fundamental premise of not leaving toxic residues in the environment. The estimated cost to create this prototype is approximately US$200,00 taking into account the individual cost of the components (Laser – US$ 100,00, H-Bridge Driver – US$ 8,00, Processing Unit US$ 20,00, Mechanisms and motors – US$ 70,00); however, this may change with further development. Importantly, the cost of commercial equipment typically decreases over time due to technological amortization. Furthermore, the physical control mechanism can be adapted for various tick genera and species, making the prototype potentially applicable to different production.

The Brazilian livestock sector raises and uses approximately 212 million cattle (IBGE, 2023), making it the second largest cattle herd in the world. However, livestock productivity is reduced by losses caused by ticks. As the main inference of this work, we envisioned that this method of physical control of ticks can significantly reduce the establishment of larvae in cattle, as they lose their motility, enhancing integrated control procedures for these ectoparasites. In this case, a new methodology or agricultural practice must be developed to control immature forms of ticks still in the environment.

Its use in animals also has potential, as it may inhibit the fixation of larvae that are moving around the host; however, despite the calculation of the laser power used, which is theoretically not able to affect the skin of the animals, further studies of damage to cattle skin will be carried out in future studies.

## Conclusion

It was possible to demonstrate that the mobility of immature forms of *R. microplus* can be inhibited or even rendered impossible through the use of low-power laser-emitting equipment (physical control), demonstrating the potential use of this technology for tick control in the near future.
